# Astragalus Polysaccharide Protect against Cadmium-Induced Cytotoxicity through the *MDA5/NF-κB* Pathway in Chicken Peripheral Blood Lymphocytes

**DOI:** 10.3390/molecules22101610

**Published:** 2017-09-25

**Authors:** Wanqiu Xie, Ming Ge, Guangxing Li, Linan Zhang, Zequn Tang, Ruyue Li, Ruili Zhang

**Affiliations:** 1 College of Veterinary Medicine, Northeast Agricultural University, Harbin 150030, China; qiuqiutangxte@163.com (W.X.); neaugeming@163.com (M.G.); liguangxing1968@hotmail.com (G.L.); 18945039894@163.com (L.Z.); tzqun1993@163.com (Z.T.); m15546091068@163.com (R.L.); 2 Heilongjiang Key Laboratory for Laboratory Animals and Comparative Medicine, Northeast Agricultural University, Harbin 150030, China

**Keywords:** cadmium, APS, chicken PBLs, *MDA5/NF-κB* signaling pathway, antioxidant function, ultrastructural pathological changes

## Abstract

Cadmium (Cd) is a known environmental pollutant that is associated with inflammation, oxidative stress, and cell apoptosis. Astragalus polysaccharide (APS) is a major component of *Astragalus membranaceus*, a vital qi-reinforcing herb medicine with favorable immuneregulation properties. To study the effect of APS on the inhibition of the cadmium-induced injury of peripheral blood lymphocytes (PBLs) in chickens through the *MDA5/NF-κB* signaling pathway, PLBs acquired from 15-day-old chickens were divided into control group, Cd group, APS + Cd group, anti-MDA5 mAb + Cd group, BAY 11-7082 (a nuclear factor kappa-light chain-enhancer of activated B cells [*NF-κB*] inhibitor) +Cd group, APS group, anti-MDA5 mAb group, and BAY 11-7082 group. The transcription levels of melanoma differentiation-associated gene 5 (*MDA5*), interferon promoter-stimulating factor 1 (*IPS-1*), *NF-κB*, and inflammatory factors tumor necrosis factor (*TNF*)-α, interleukin (*IL*)-1β, and *IL-6* were measured by quantitative real-time PCR. MDA5 protein expression was measured by western blotting. Levels of malondialdehyde (MDA), glutathione peroxidase (GSH-Px), and superoxide dismutase (SOD) were measured by corresponding antioxidant kit. The morphological change of PBLs was measured by transmission electron microscopy. The results showed that Cd significantly increased the expression of *MDA5*, *IPS-1*, *NF-κB*, and their downstream cytokines, *IL-1β* and *TNF-α*, *IL-6* in PLBs. In addition, a high level of MDA was observed in the Cd treatment group; the activities of GSH-Px and SOD were significantly lower in the Cd treatment group than those in controls (*p* < 0.05). Ultrastructural changes of PBLs showed that Cd promoted autophagy, apoptosis, and necrosis in PBLs. However, APS can efficiently improve Cd-induced cell damage by decreasing the activation of the *MDA5* signaling pathway. The effect is consistent with that of anti-MDA5 mAb or/and BAY. The results indicated that APS inhibited Cd-induced cytotoxicity through the regulation of MDA5/NF-κB signaling.

## 1. Introduction

Cadmium (Cd), a transition metal, is found widely in the environment, having a significant effect because of its toxicity on human and animal health [1]. Cd can be absorbed into the human body via food chains or through acute or chronic occupational and environmental exposures, resulting in the occurrence of many diseases, even cancers [2], which is retained within the body with little excretion, and when there is an accumulation of the metal within the vital organs, it may produce toxic effects in the kidneys, liver, lungs [3], cardiovascular system, immune system [4], and reproductive system [5,6]. The immune system is a target system for Cd toxicity [7]. Pathak, N. et al. showed that Cd exposure reduced the cell viability of lymphocytes in rodents and humans, and also induced oxidative stress, apoptosis, and necrosis [8]. Another study suggested that Cd was capable of inducing apoptosis in murine thymocytes, which is accompanied by a loss in cell viability, significant DNA fragmentation, increased reactive oxygen species (ROS), and mitochondrial membrane depolarization [7]. Furthermore, Björn Fagerberg et al. imply that Cd is a possible cause for the increased levels of inflammatory markers and future cardiovascular disease [9].

The first line of host defense is innate immunity. The innate immune system recognizes pathogen-associated molecular patterns through pattern-recognition receptors (PRRs). PRRs consist of Toll-like receptors (TLRs), nucleotide-binding oligomerization domain protein-like receptors, and retinoic acid-inducible gene I-like receptors (RLRs) [10]. In the RLR family, *MDA5* recognizes viral RNAs in the cytoplasm through C-terminal DExD/H box RNA helicase, and the two CARDs function through CARD-CARD interacts with interferon promoter-stimulating factor 1 (*IPS-1*), leading to the activation of interferon regulatory factor 3 and *NF-κB* [11], which finally induces type I interferon (*IFN-I*) production and triggers a series of inflammatory reactions such as *TNF-α*, *IL-1β*, and *IL-6*. The preliminary study created a chicken-based model for exposure to Cd in vivo, which showed that the expression of *MDA5* and its signaling pathway molecule were enhanced in the model group [12]. These results suggest that the *MDA5* signaling pathway may be involved in the injury process induced by Cd. However, further studies are needed to confirm this.

*Astragalus membranaceus*, known as Huang Qi, is one of the most commonly used antiaging herbs in traditional Chinese medicine. Astragalus polysaccharide (APS) is a major active ingredient of *Astragalus membranaceus* [13]. The anti-oxidation and anti-inflammatory properties of Astragalus polysaccharide (APS), as well as its protective effects on various organs, have been investigated extensively [14]. Astragalus polysaccharide can attenuate lipopolysaccharide-induced inflammatory responses in microglial cells [15]. APS possesses multiple pharmacological and immunomodulatory activities. It plays an important role in disease therapy by regulating the functions of immune cells and the expression of cytokines, increasing activity of antioxidant enzymes, and reducing lipid peroxidation, etc. [16,17]. Studies have reported APS ameliorates ionizing radiation-induced oxidative stress in mice [18]. Currently, there are no reports about the effect of APS on Cd poisoning in animals. APS exerts immunomodulatory effects via *TLR4*-mediated *MyD88*-dependent signaling pathway in vitro and in vivo [19]. Hence, our study proposes that APS inhibited Cd-induced cytotoxicity through the regulation of *MDA5/NF-κB* signaling.

In summary, this study was based on Cd exposure [20] in peripheral blood lymphocytes (PBLs) of normal chickens in vitro, and incubated APS [21] in PBLs. By detecting the expression of MDA5 and its signal pathway molecule, the content of cytokines, antioxidant function changes, and morphology injuries can be used to study the effect and mechanism of APS on Cd-induced cytotoxicity in chickens PBLs.

## 2. Results

### 2.1. Cell Viability

**Figure 1:**
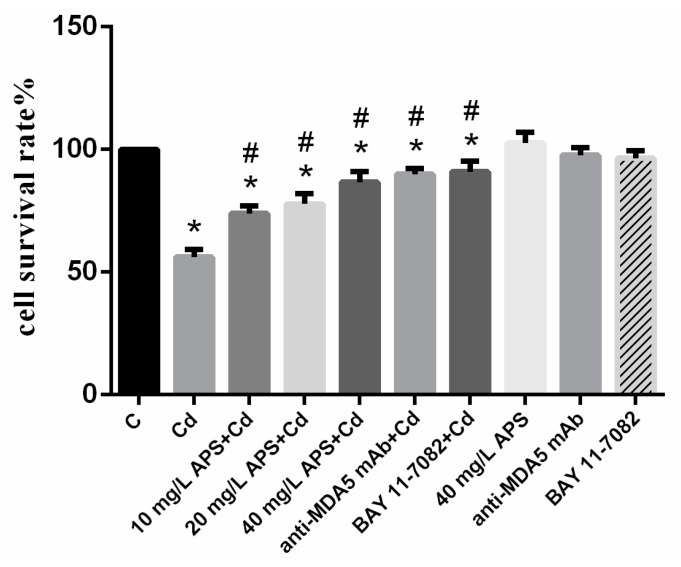
Cell viability of peripheral blood lymphocytes (PBLs). Each value is presented as mean ± standard deviation of three individuals. * *p* < 0.05 compared with the control group; ^#^ *p* < 0.05 compared with the Cd group.

The cell viability (Figure 1) was significantly decreased in the Cd group as compared with that in the control group (*p* < 0.05). However, the downregulation could be inhibited by APS supplementation in a dose-dependent manner. Similarly, the cells pretreated with anti-MDA5 mAb and/or NF-κB inhibitor BAY produced analogous effects with the APS + Cd group. However, the use of APS, anti-MDA5 mAb, or BAY alone had no significant effects on cell viability (*p* > 0.05). One concentration of APS (40 mg/L) was used for further experiments.

### 2.2. Expression of MDA5

**Figure 2:**
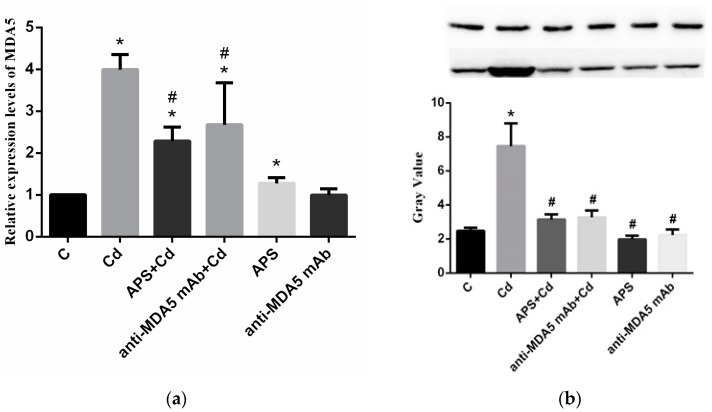
The mRNA expression of *MDA5* (**a**) and the protein expression of MDA5 (**b**) in PBLs. Each value is presented as mean ± SD of three individuals. * *p* < 0.05 compared with the control group; ^#^ *p* < 0.05 compared with the Cd group.

As illustrated in Figure 2a, the results showed that the mRNA level of *MDA5* in PBLs was measured by quantitative real-time PCR. In the current study, the Cd group expression of *MDA5* showed an elevation in PBLs when compared with those of the control group (*p* < 0.05). The expression of *MDA5* in the APS + Cd group was lower than that in the Cd group but higher than that in the control group (*p* < 0.05). The effects of cells pretreated with anti-MDA5 mAb were similar with the APS + Cd group (*p* < 0.05). However, the treatment of APS caused a mild increase in the activity of the expression of *MDA5* (*p* < 0.05) and the expression of *MDA5* in the anti-MDA5 mAb group showed nonsignificance with the control group (*p* > 0.05).

The MDA5 protein expression in PBLs was measured by Western blot analysis (Figure 2b). As shown in the figures, the protein expression of MDA5 was elevated in the Cd group (*p* < 0.05), whereas the APS + Cd group showed significantly lower MDA5 protein expression than the Cd group (*p* < 0.05). The effect of cells pretreated with anti-MDA5 mAb was similar in the APS + Cd group (*p* < 0.05). The difference between the control group and the APS group and anti-MDA5 mAb group was not significant.

### 2.3. mRNA Levels of MDA5’s Downstream Signaling Molecule

**Figure 3:**
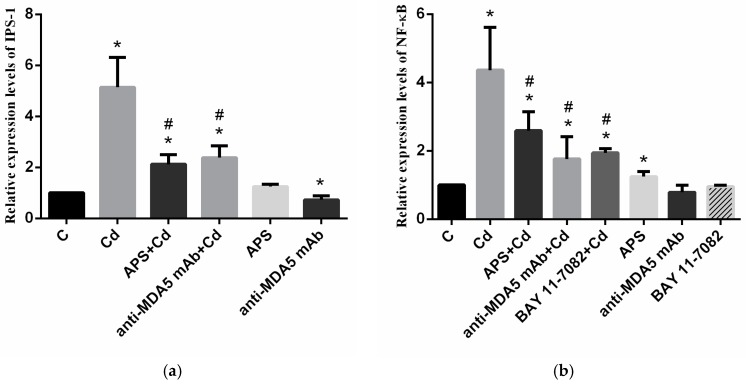
The mRNA levels of *MDA5* signaling pathway-related genes in PBLs. (**a**) The mRNA level of *IPS-1* in PBLs; (**b**) The mRNA level of *NF-κB* in PBLs. Each value is presented as the mean ± SD of three individuals. * *p* < 0.05 as compared with the control group; ^#^ *p* < 0.05 as compared with the Cd group.

To further study the *MDA5* signaling pathway, we measured the relative mRNA levels of *IPS-1* and *NF-κB* in this signaling pathway. As illustrated in Figure 3, the mRNA levels of *IPS-1* and *NF-κB* were significantly higher in the Cd group than in the control group (*p* < 0.05), However, the mRNA level of *IPS-1* was noticeably lower in the APS + Cd and anti-MDA5 mAb + Cd groups. The mRNA level of *NF-κB* was noticeably lower in the APS + Cd, BAY + Cd, and anti-MDA5 mAb + Cd groups than in the Cd group (*p* < 0.05). In the APS group, the *IPS-1* level showed no significant difference when compared with that in the control group (*p* > 0.05), and the expression of *NF-κB* was significantly higher than that in the control group (*p* < 0.05). In the anti-MDA5 mAb group, the *NF-κB* level showed no significant difference as compared with that in the control group (*p* > 0.05), and the expression of *IPS-1* was significantly lower than that in the control group (*p* < 0.05). In the BAY group, the *NF-κB* level showed no significant difference compared with that in the control group (*p* > 0.05).

### 2.4. mRNA Levels of Cytokines

**Figure 4:**
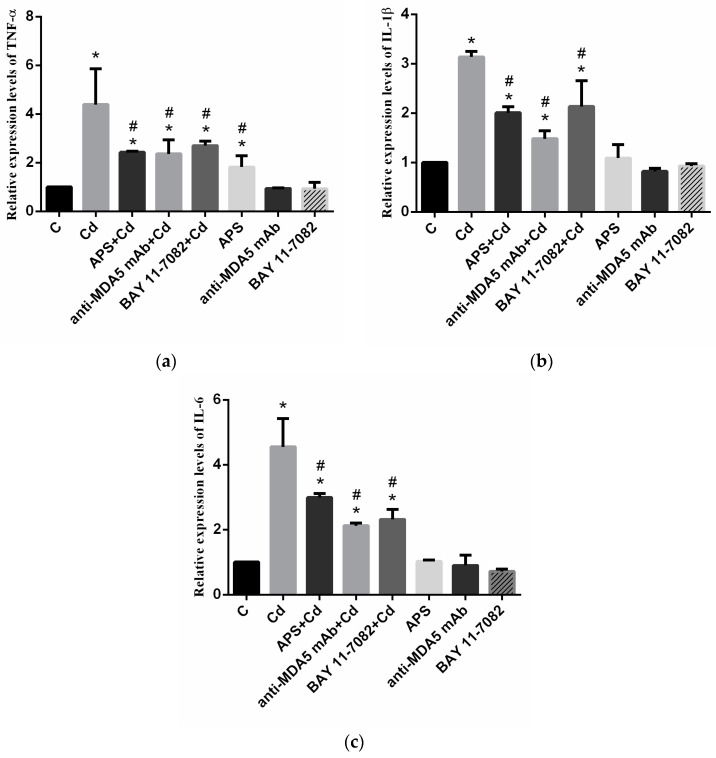
The mRNA levels of cytokines in PBLs. (**a**) The result of mRNA level of *TNF-α* in PBLs; (**b**) The mRNA level of *IL-1β* in PBLs; (**c**) The mRNA level of *IL-6* in PBLs. Each value is presented as mean ± SD of three individuals. * *p* < 0.05 compared with the control group; ^#^ *p* < 0.05 compared with the Cd group.

The mRNA levels of *TNF-α*, *IL-1β* and *IL-6* are shown in Figure 4a,c, respectively. The expression levels of *TNF-α*, *IL-1β* and *IL-6* were significantly increased in the Cd group when compared with those in the control group (*p* < 0.05). The expression levels of *TNF-α*, *IL-1β*, and *IL-6* were significantly decreased in the APS + Cd group compared with that in the Cd group (*p* < 0.05). The mRNA levels of the three inflammatory factors were also noticeably lower in the BAY + Cd and anti-MDA5 mAb + Cd groups than in the Cd group (*p* < 0.05). The expression of *IL-6* and *IL-1β* showed no significant difference in the APS group (*p* > 0.05), whereas there was significant upregulation in the level of *TNF-α* in the APS group when compared with the control group (*p* < 0.05). In the anti-MDA5 mAb group and the BAY group, the expression of the three inflammatory factors showed no significant difference when compared with that in the control group (*p* > 0.05).

### 2.5. Antioxidant Activity

**Figure 5:**
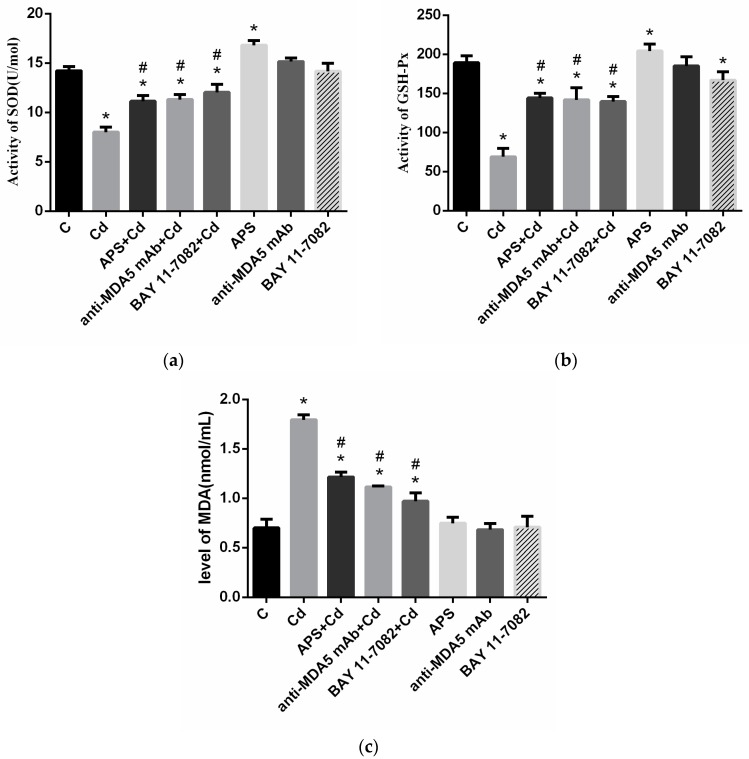
The antioxidant activity of PBLs. (**a**) The activity of superoxide dismutase (SOD) in PBLs; (**b**) The activity of GSH-Px in PBLs; (**c**) The content of malondialdehyde (MDA) in PBLs. Each value is presented as mean ± SD of three individuals. * *p* < 0.05 as compared with the control group; ^#^ *p* < 0.05 as compared with the Cd group.

As illustrated in Figure 5a,b, the antioxidant index detection results imply that the treatment of APS caused a mild increase in the activity of the SOD and the GSH-Px (*p* < 0.05), but the Cd group showed a marked decline in the activity in PBLs (*p* < 0.05). Pretreatment of APS caused a significant recovery in the activity when compared with that of the SOD and the GSH-Px in the Cd group (*p* < 0.05). The effects of the pretreatment of BAY and anti-MDA5 mAb showed the same consequence with the APS + Cd group (*p* < 0.05). In the anti-MDA5 mAb group, there were no significant differences in the activity of SOD and GSH-Px when compared with that in the control group (*p* > 0.05). In the BAY group, there were no significant differences in the activity of SOD (*p* > 0.05), but the activity of GSH-Px were significantly decreased as compared with that in the control group (*p* < 0.05). An estimation of malondialdehyde (MDA) (Figure 5c) was carried out to assess the extent of lipid peroxidation in PBLs after treatment. Cd demonstrated an increase in the MDA level in PBLs (*p* < 0.05). In the combination group (APS + Cd), the MDA levels decreased in the samples (*p* < 0.05), and the effects of the pretreatment of BAY and anti-MDA5 mAb showed the same results with the APS + Cd group, indicating the ameliorative effect of APS on Cd toxicity. However, the use of APS, BAY, or anti-MDA5 mAb alone had no significant effects on the MDA level.

### 2.6. Ultrastructural Pathological Changes

**Figure 6:**
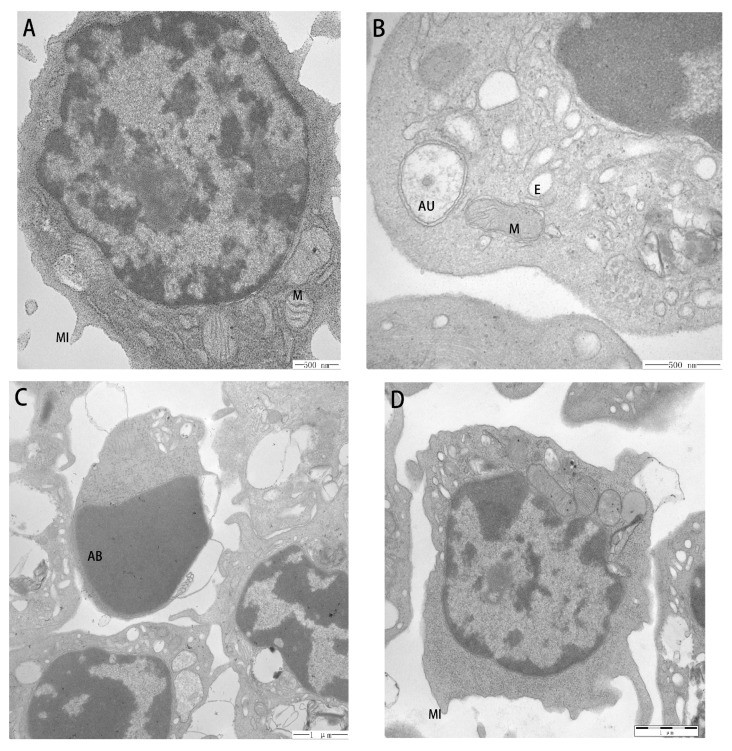
Ultrastructural pathological changes of PBLs. (**A**) The control group. The condensed chromatin abutted the nuclear membrane. The mitochondria (M) and other organelles were intact. Some microvilli (MI) distributed in the surface of cells; (**B**) The Cd group. The mitochondrial crista broke or dissolved and the endoplasmic reticulum (E) expanded. In addition, the autophagosomes (AU) appeared; (**C**) The Cd group. The apoptotic bodies (AB) appeared; (**D**) The APS + Cd group. The MI structure of the cell surface was a bit unclear and the cell surface was a little smooth.

Electron microscopy showed a loose chromatin of the PBLs in the control group, with an even distribution in the nucleus, cytoplasm light staining, and some microvilli distributing in the surface (Figure 6A). In the Cd group, the microvilli disappeared in the early stage of apoptosis; there was concentration in the nucleus, the chromatin was condensed to the periphery of the nuclear membrane; and, the mitochondrial crista broke or dissolved and the endoplasmic reticulum expanded. Finally, apoptotic bodies and autophagosomes appeared (Figure 6B,C). When compared with the Cd group, the cellular morphology in the APS + Cd group improved significantly, and the chromatin appeared a little loose. However, the microvilli structure of the cell surface was still unclear, and the cell surface was smoother than that of the control group. In addition, the expansion of endoplasm and fracture of mitochondrial crista were reduced in the APS + Cd group when compared with that in the Cd group, but still existed (Figure 6D).

## 3. Discussion

Long-term exposure of animals to Cd induces lesions in many organs, including organs involved in the immune response. Studies have shown that the immune system is highly sensitive to Cd [22]. Many studies have demonstrated that Cd can impair the antioxidant system of both animals and plants [23,24], and cause macromolecules that cause damage, such as mutations in DNA and the destruction of protein structure and function. Increased superoxide radicals, lipid peroxidation, and alterations in gene expression may lead to apoptosis. APS from natural sources is a class of macromolecules that can profoundly affect the immune system and therefore have the potential as immunomodulators with wide clinical applications [25,26]. In addition, APS can eliminate ROS, inhibit mitochondrial injury, and enhance the activity of antioxidant enzymes [27]. In the present study, we examined the effect of supplemented APS on Cd-induced cytotoxicity in chicken PBLs. The protective effect of ABP under Cd-induced cytotoxicity could be explained by three reasons. First, APS ameliorated the Cd-induced structural damage and cell vitality damage in chicken PBLs, as reflected by changes in histology and MTT. Second, APS mitigated Cd-induced oxidative damage. Finally, the immuneregulating effect of APS mitigated the Cd-induced inflammatory response. These are closely related to the activation of the *MDA5* signaling pathway.

Yang SH et al. indicated that the number of apoptotic cells in hen ovaries increased in the Cd-alone-treated group, and extensive damage was observed in the ovaries [5]. Some studies have demonstrated that Cadmium induced autophagy through ROS-dependent activation of the *LKB1–AMPK* signaling in skin epidermal cells [28]. In this study, an experiment with MTT showed that in the Cd group, viability of PBLs was significantly reduced. Electron microscopy showed that in the Cd group, the morphological changes of necrosis, autophagy, and apoptosis in PBLs were observed, indicating that Cd induced damnification of the PBLs. Some studies have demonstrated that APS can inhibit autophagy and apoptosis from peroxide-induced injury in C_2_C_12_ myoblasts [29]. In this study, preincubation of APS significantly inhibited the decrease of Cd-induced cell viability. Electron microscopy showed that the morphology of PBLs in APS + Cd group was significantly improved when compared to that in the Cd group. This vast amount of evidence confirms that APS has a significant protection effect on Cd-induced reduction of cell activity and the damnification of PBLs. Continuously, the exact mechanism requires further study.

As a stressor, heavy metals like Cd can upset the balance of the oxidant/antioxidant system by affecting the regulation of enzymatic oxidation, protein oxidation, and lipid peroxidation activities, and cause tissue damage [30]. Cd-induced toxicity in living systems may be caused by an increase in lipid peroxidation, which could be accredited to changes in antioxidant defense systems, including the enzymes thioredoxin reductase, glutathione peroxidase (GPx), and reduced glutathione (GSH), which generally offers protection to living systems from toxicity due to free radicals and protects the cells from the damage of ROS [31]. Moreover, the antioxidant enzymes superoxide dismutase (SOD) is the first step of the antioxidant pathway, which dismutates O^2−^ to H_2_O_2_ [32]. Hence, their levels were estimated in PBLs to assess the burden of oxidative stress. Moreover, MDA is an oxidized lipid metabolite and can be used to measure the level of oxidative stress in an organism [8]. Some studies have reported that Cd accumulation results in a decrease in antioxidant enzymes activities and an increase in the MDA level in both plasma and tissues [33]. Previous studies have connected Cd with oxidative stress [34,35]. Babatunji et al. showed that a high level of malondialdehyde (MDA) and ROS production was observed in the Cd treatment group of chicken splenic lymphocytes; the activities of GSH-Px and SOD were significantly lower in the Cd treatment group than those in controls in chicken splenic lymphocytes [36], which indicated that Cd was able to alter the antioxidant defense system. Studies have reported APS can attenuate iron overload-induced dysfunction of mesenchymal stem cells via suppressing mitochondrial ROS [37], indicating that APS has a resistance to metal-induced oxidative damage. Currently, there are no reports about the effect of APS on Cd poisoning in animals. In this study, the MDA level decreased but the activities of antioxidant enzymes (SOD and GSH-Px) increased in the combination group (APS + Cd), indicating that APS can not only significantly increase the activity of SOD and GSH-Px in PBLs of chickens, but can also reduce the harmful effects of oxidants. In the present study, we demonstrated that APS played a protective role in Cd-induced oxidative dysfunctions. However, further studies are needed to address whether other mechanisms play roles in APS against Cd-induced oxidative damage.

Metals such as Cd can damage numerous biochemical pathways [38]. Lee et al. indicated that metals induced many types of protein kinases via the signal transduction within cells, including critical kinases mitogen-activated protein kinases (MAPKs), phosphatidylinositol 3-kinase *(PI3K)/Akt* pathway and Rous sarcoma oncogene cellular homolog (Src) [39]. APS showed an antioxidant effect in response to palmitate stimulation in skeletal muscle cells through a mechanism involving the activation of the *ROS-ERK-NF-κB* pathway [40]. However, no studies have shown that APS is resistant to oxidative stress through the *MDA5* signaling pathway. In order to determine whether APS protected against the Cd-induced oxidative stress via *MDA5* signaling pathway in the present study, the cells were treated with antibody against MDA5 and NF-κB inhibitor (BAY) against *NF-κB* prior to Cd treatment. We found that the SOD and GSH-Px activities in anti-MDA5 mAb + Cd group and BAY + Cd group on PBLs were significantly reduced when compared with Cd group, which was identical to the results of APS + Cd group, suggesting that APS had the capacity to protect against the Cd-induced oxidative stress through *MDA5/NF-κB* signaling pathway.

In animals, the induction of the expression of cytokines by the environmental chemicals is considered as an inflammation response, which is a key mediator of the host response against microbial pathogens [39,41]. Hyun et al. demonstrated that Cd induced *IL-8* production through *NF-κB* activation in the human intestinal epithelial cell, Caco-2 [42]. Fengping Xu et al. proved that Cd significantly increased the expression of *IL-1β*, *TNF-α*, and other cytokines in chicken spleen lymphocytes [43]. There is evidence supporting the anti-inflammatory effect and favorable immuneregulating effects of APS in various experimental models in vivo and in vitro [15,44]. Research has demonstrated that APS administration inhibits the expression of pro-inflammatory genes, such as *IL-1β* and *IL-6* in palmitate-treated RAW264.7 cells in vitro [14]. In this study, we demonstrated that the expression of *TNF-α*, *IL-1β*, and *IL-6* were significantly increased in the Cd group when compared with that in the control group. However, the expression levels of *TNF-α*, *IL-1β*, and *IL-6* were significantly decreased in the APS + Cd group as compared to that in the Cd group, which demonstrated that APS was able to prevent the Cd-induced production of inflammatory mediators. This is the first report demonstrating that APS is capable of regulating Cd-stimulated inflammatory mediator production in PBLs. However, further studies are needed to address whether other mechanisms play roles in ABP against Cd-induced inflammatory injury.

Cd is a potent immunotoxicant, which has been reported to affect the immunocytes and immune organs both in humans and in rodents [45]. Researches indicated that Cd exposure dramatically enhanced mRNA level of *TNF-α* via the master inflammatory regulator—*NF-κB* in the brain, ovary and liver of zebrafis [46,47]. *NF-κB* is widely distributed in biological organisms that participate in the regulation of multiple genes, inflammatory response and immune response and is essential in priming inflammasome activation for production of cytokines like *TNF-α*, *IL-6* and also of pro-*IL-1β* [48,49]. Researches indicated that the *MDA5* gene could interacte with *IPS-1* to activate the downstream gene *IRF3* and *NF-κB* and influenced the production of type I interferon and inflammatory cytokines [50]. In this study, the Cd group mRNA expression of *MDA5* and *MDA5* downstream signaling molecules (*IPS-1* and *NF-κB*) showed an increase in PBLs when compared with those of the control group. However, the mRNA expression levels of *MDA5*, *IPS-1*, and *NF-κB* were significantly decreased in the anti-MDA5 mAb + Cd group as compared to that in the Cd group (*p* < 0.05), indicating that Cd activates the MDA5 signaling pathway in PBLs of chickens. Further illustrated this mechanism, the inhibitor of *NF-κB*, BAY 11-7082 tests were supplemented. The results pretreatment with BAY 11-7082 showed the inhibition effects of Cd-induced production of cytokines by suppressing the *NF-κB* signaling pathway, indicating that Cd can induce immune imbalance in chicken PBLs via the *MDA5/NF-κB* signaling pathway. However, currently, there are no reports on the immunoregulatory effect of APS on the *MDA5* signaling pathway. Researches showed that APS could suppress the production of *TNF-α* and *IL-1β* by LPS stimulated macrophages by inhibiting *MAPK/NF-κB* pathway [17]. Other research shows that APS exerts immunomodulatory effects via the *TLR4*-mediated *MyD88*-dependent signaling pathway in vitro and in vivo. Both *MDA5* and *TLR4* belong to the natural pattern recognition receptors. In the present study, we studied that the mRNA expression levels of *MDA5*, *IPS-1*, and *NF-κB* were significantly decreased in the APS + Cd group as compared to that in the Cd group (*p* < 0.05). Continuously, the cells pretreated with *NF-κB* inhibitor BAY or/and anti-MDA5 mAb produced analogous effects with the APS + Cd group. As a consequence, we demonstrate that APS plays an immuneregulating effect against Cd-induced immune imbalance in chicken PBLs via the *MDA5/NF-κB* signaling pathway.

## 4. Materials and Methods

### 4.1. Preparation of Chicken PBLs and Treatment

Ethical treatment of animals used in this study was approved by the Animal Welfare Committee protocol (#NEAU-2013-02-0252-11) at Northeast Agricultural University (Harbin, China) Anticoagulant blood samples were dissected from 15-day-old Hyline egg-laying chickens (Harbin, China), and carefully placed in a tube containing lymphocyte in a separation medium (Tian Jin Hao Yang Biological Manufacture Co. Ltd., Tianjin, China), and the specific extraction method of chicken PBLs was routine procedure in our laboratory [12]. The acquired cells were cultured in a 37 °C 5% CO_2_ incubator for 24 h. The APS were extracted from Astragalus by the Sevag method according to our previous study [21]. To monitor various parameters in the current investigation, the specific groups and treatments are shown in Table 1.

### 4.2. MTT Assay

Cell viability assay was performed by using 3-(4,5-dimethyl-2-yl)-2,5-diphenyl tetrazolium bromide (MTT) reduction method, as described earlier, with slight modifications. The PBLs were seeded in 96-well plates with the density of 1 × 10^5^ cells/mL, and the PBLs of all groups were incubated in a 37 °C 5% CO_2_ incubator for 24 h. 10 μL MTT (5 mg/mL, Sigma-Aldrich, St. Louis, MO, USA) was added to the wells at 4 h and again when incubated. The plate was centrifuged at 1200× *g* for 10 min, and 100 μL dimethyl sulfoxide was added after removing the supernatant to dissolve the formed formazan. After gentle shaking for 5 min, the optical density (OD) at a wavelength of 570 nm was measured using an enzyme-linked immunosorbent assay plate reader (Bio-Rad, Hercules, CA, USA).

### 4.3. Quantitative Real-Time PCR

Total RNA was extracted from PBLs according to the specification of the RNA quick extraction kit (BioTeke Corporation, Beijing, China). The complementary DNA was then synthesized. The primers used are shown in Table 2. The messenger RNA (mRNA) levels of *MDA5*, *IPS-1*, *NF-κB*, *IL-1β*, *IL-6*, and *TNF-α* were measured using the premixed dye of Power SYBR real-time PCR (BioTeke Corporation, China) and the Light Cycler 480 real-time PCR (Roche, Basel, Switzerland); each sample’s reaction volume was 20 μL. The reaction conditions were 1 cycle at 95 °C for 2 min, and 40 cycles at 95 °C for 20 s, 60 °C for 20 s, and 72 °C for 30 s. All of the data from the quantitative real-time PCR experiments were analyzed by the 2^−ΔΔ*C*t^ method.

### 4.4. Western Blotting Analysis

Total protein was extracted from PBLs. The PBLs of each group were resuspended in 300 μL radioimmunoprecipitation assay tissue/cell lysis solution (Beyotime Institute of Biotechnology, Haimen, China), shaken, and rested for 4 min, and then centrifuged at 12,000 rpm at 4 °C for 5 min to obtain the protein. The protein samples were separated using 8% sodium dodecyl sulfate polyacrylamide gel electrophoresis and then transferred to nitrocellulose membrane. The membrane was blocked using skim milk (5%) and then incubated with the primary antibody of MDA5 [12] (1:1000) at 4 °C overnight, followed by incubation with horseradish peroxidase-conjugated goat anti-mouse immunoglobulin (Ig)G (1:5000, Beijing Solarbio Science & Technology Co., Ltd., Beijing, China) for 1 h after washing three times. At the same time, the membrane was incubated with the β-actin antibody (1:1000, Beijing Zhong Shan-Golden Bridge Biological Technology Co., Ltd., Beijing, China) followed by incubation with horseradish peroxidase-conjugated goat anti-mouse IgG (1:5000). Positive signals were detected with the ChemiScope 3011 chemiluminescence system (Clinx Science Instrument Co., Ltd., Shanghai, China). The integrated density (IntDen) of each band was analyzed by ImageJ software (National Institutes of Health, Bethesda, MD, USA), and the protein expression of MDA5 was expressed by the ratio of the IntDen of MDA5 to the IntDen of β-actin.

### 4.5. Measurement of Oxidative Stress

The content of MDA and the activities of SOD and GSH-Px were assayed using kits following the manufacturer’s instructions (Nanjing Jiancheng Bioengineering Institute, Nanjing, China).

### 4.6. Morphological Study

PBLs were extracted as described previously. The PBLs of the control group, the Cd group, and the APS+Cd group cells were fixed, respectively, by 2.5% glutaraldehyde and 1% osmic acid at 4 °C. After being washed in 0.1 M phosphate buffered saline (pH 7.4), dehydrated routinely, embedded in Epon812, and polymerized at 80 °C for 48 h, the PBLs were sliced with a Leica ultramicrotome (Leica, Wetzlar, Germany). Ultrathin sections were stained with uranyl acetate and lead citrate, and were screened at 80 kV with a transmission electron microscope (Hitachi H-7650, Tokyo, Japan).

### 4.7. Statistical Analysis

Statistical analysis data were evaluated with the SPSS 19.0 software (SPSS Inc., Chicago, IL, USA) and GraphPad Prism 6 (GraphPad Inc., La Jolla, CA, USA). Statistical comparisons were made using one-way analysis of variance. A value of *p* < 0.05 was considered as statistically significant.

## 5. Conclusions

APS can efficiently inhibit Cd-induced immune imbalance in chicken PBLs through the regulation of *MDA5/NF-κB* signaling, and it can decrease Cd-induced expression of cytokines (*IL-1β*, *IL-6*, and *TNF-α*). In addition, APS can significantly enhance Cd-induced antioxidant enzyme (SOD, GSH-Px) activity and reduce the Cd-induced MDA level through the regulation of *MDA5/NF-κB* signaling. Ultimately, APS can improve Cd-induced decrease in cell viability and damage to cell morphology. Thus, APS has a protective effect on Cd-induced cytotoxicity in chicken PBLs through the *MDA5/NF-κB* pathway.

## Figures and Tables

**Table 1:** The group and treatment of chicken PBLs.

Group	Treatment
control group	no treatment
Cd group	incubating with CdCl_2_ (10^−6^ mol/L, Guangfu Technology Co,. Ltd., Tianjin, China) for 24 h
APS + Cd group	incubating with APS (40 mg/L) for 2 h first, then adding CdCl_2_ (10^−6^ mol/L) for 24 h
anti-MDA5 mAb + Cd group	incubating with anti-MDA5 mAb (5 mg/L) for 2 h first, then adding CdCl_2_ (10^−6^ mol/L) for 24 h
BAY + Cd group	incubating with BAY11-7082 (10^−4^ mol/L, Beyotime, Haimen, China) for 2 h first, then adding CdCl_2_ (10^−6^ mol/L) for 24 h
10 mg/L APS group	incubating with 10 mg/L APS for 24 h
20 mg/L APS group	incubating with 20 mg/L APS for 24 h
40 mg/L APS group	incubating with 40 mg/L APS for 24 h
anti-MDA5 mAb group	incubating with anti-MDA5 mAb (5 mg/L) for 24 h
BAY group	incubating with BAY11-7082 for 24 h

**Table 2:** Gene specific primers used in the quantitative real-time PCR.

Primer Name		Primer Sequence	Primer Length (bp)
*MDA5*	Forward	TCAGGAGGAGGACGACCACGAT	22
	Reverse	TTCCCACGACTCTCAATAACAG	22
*IPS-1*	Forward	TGCAGGGAGGCCATACACCAGTG	23
	Reverse	TCCACCTCCCAAGGTGACCCGTG	23
*NF-κB*	Forward	TCTGAACAGCAAGTCATCCATAACG	25
	Reverse	AAGGAAGTGAGGTTGAGGAGTCG	23
*IL-1β*	Forward	TTCCGCTACACCCGCTCACAGT	22
	Reverse	CCGCTCATCACACACGACAT	20
*IL-6*	Forward	ATGGTGATAAATCCCGATGAAG	22
	Reverse	CCTCACGGTCTTCTCCATAAAC	22
*TNF-α*	Forward	CAGATGGGAAGGGAATGAAC	20
	Reverse	AGAGCATCAACGCAAAAGGG	20
*β-actin*	Forward	ATTGCTGCGCTCGTTGTT	18
	Reverse	CTTTTGCTCTGGGCTTCA	18
